# Conserved epitopes of influenza A virus inducing protective immunity and their prospects for universal vaccine development

**DOI:** 10.1186/1743-422X-7-351

**Published:** 2010-11-30

**Authors:** Zuzana Staneková, Eva Varečková

**Affiliations:** 1 Institute of Virology, Slovak Academy of Sciences, Dúbravská cesta 9, 845 05 Bratislava, Slovak Republic

## Abstract

Influenza A viruses belong to the best studied viruses, however no effective prevention against influenza infection has been developed. The emerging of still new escape variants of influenza A viruses causing epidemics and periodic worldwide pandemics represents a threat for human population. Therefore, current, hot task of influenza virus research is to look for a way how to get us closer to a universal vaccine. Combination of chosen conserved antigens inducing cross-protective antibody response with epitopes activating also cross-protective cytotoxic T-cells would offer an attractive strategy for improving protection against drift variants of seasonal influenza viruses and reduces the impact of future pandemic strains. Antigenically conserved fusion-active subunit of hemagglutinin (HA2 gp) and ectodomain of matrix protein 2 (eM2) are promising candidates for preparation of broadly protective HA2- or eM2-based vaccine that may aid in pandemic preparedness. Overall protective effect could be achieved by contribution of epitopes recognized by cytotoxic T-lymphocytes (CTL) that have been studied extensively to reach much broader control of influenza infection. In this review we present the state-of-art in this field. We describe known adaptive immune mechanisms mediated by influenza specific B- and T-cells involved in the anti-influenza immune defense together with the contribution of innate immunity. We discuss the mechanisms of neutralization of influenza infection mediated by antibodies, the role of CTL in viral elimination and new approaches to develop epitope based vaccine inducing cross-protective influenza virus-specific immune response.

## 1. Introduction

Influenza remains a serious respiratory disease in spite of the availability of antivirals and inactivated trivalent vaccines, which are effective for most recipients. Influenza viruses are RNA viruses with strongly immunogenic surface proteins, especially the hemagglutinin. Error-prone RNA-dependent RNA polymerase and segmented genome enable influenza viruses to undergo minor (antigenic drift) as well as major (antigenic shift) antigenic changes, which permit the virus to evade adaptive immune response in a variety of mammalian and avian species, including humans. The unpredictable variability of influenza A viruses, which cause yearly epidemics in human population, is the main reason why no effective prevention against influenza infection exists up to date. Currently available vaccines induce antibodies against seasonal and closely related antigenic viral strains, but do not protect against antibody-escape variants of seasonal or novel influenza A viruses. Therefore, there is a call for development of a vaccine, which would be protective against virus strains of different HA subtypes and would not need to be updated every year. New approach to prepare a universal vaccine lies in the selection of conserved epitopes or proteins of influenza A virus, which induce cross-protective immune response, particularly M2, HA2, M1, NP [1-3].

## 2. Induction of adaptive immunity by influenza infection

**Figure 1:**
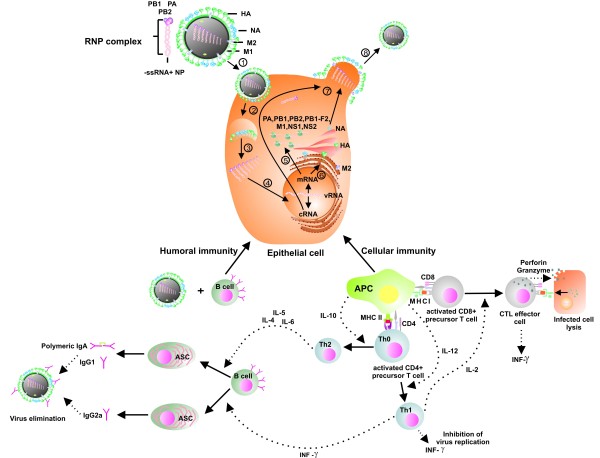
**Humoral and cellular immunity induced by influenza virus infection**. **(1) **Influenza virus binds to the receptor on the host cell and entry the cell by receptor-mediated endocytosis. **(2) **The endosomal acidification permits fusion of the host and viral membranes by altering the conformation of hemagglutinin. **(3) **Upon the fusion, viral ribonucleoprotein complexes (RNP complex) are released into the cytoplasm and **(4) **transported to the nucleus, where the viral RNAs (vRNA) are transcribed into messenger RNAs (mRNA) and replicated by the viral RNA-dependent RNA polymerase complex into complementary RNA (cRNA). **(5) **mRNA are exported to the cytoplasm for translation of structural proteins. **(6) **Synthesis of envelope proteins take place on ribosomes of endoplasmic reticulum. **(7) **The newly synthesized viral RNPs are exported from the nucleus to the assembly site at the apical plasma membrane, where **(8) **new virus particles are budding and release out of host cells. Influenza virus infection triggers innate (not shown) and adaptive immune response where the effector cells and molecules are involved in restriction of viral spread, as follows: The cellular immune response *(right) *is initiated after recognition of viral antigens presented via MHCI and MHC II molecules by antigen presenting cells (APC), which then leads to activation, proliferation and differentiation of antigen-specific CD8+ T or CD4+ cells. These cells gain effector cell function and either they help directly (Th1 or Th2 cell) to produce antibodies or, CTL effector cells recognize antigen peptides presented by MHCI on APC and kill the virus infected cells by exocytosis of cytolytic granules. The humoral immune response *(left)*is mediated by specific antibodies (e.g IgG, IgA) produced by antibody secreting plasma cells (ASC) which are the final stage of B cell development. This process is aided by CD4+ T helper and T cell-derived cytokines essential for the activation and differentiation of both B-cell responses and CD8+ T cell responses.

Influenza infection induces specific humoral immunity represented by systemic and local antibody response, as well as cellular immunity, represented by specific T-cell response (Figure 1). Both of them are important in the host defense against influenza infection, because of their close cooperation mediated by various immune mechanisms. Dendritic cells and macrophages (antigen presenting cells, APCs) play an important role in initiating and driving of adaptive immune response [4]. Exogenous viral antigens, including inactive viral particles, intact viruses or infected cells, are taken up by APCs through endocytosis or phagocytosis. Their further processing results in generation of peptides that are presented via MHC I or MHC II molecules to CD8+ precursor T-cell and CD4+ helper T-cell precursors (Th0), respectively. Th0 cells are subdivided to Th1- and Th2-type helper cells, based on the cytokine profiles they produce. Following influenza infection, APCs secrete IL-12 that contributes to the differentiation of Th0 into Th1 cells, which secrete IFN-γ and help to produce IgG2a antibodies [5,6]. Th1 cells also produce IL-2, required for the proliferation of the virus-specific CD8+ CTLs. In contrast, when IL-10 is present early in the immune response, Th0 cells differentiate to Th2 cells, which secrete IL-4, IL-5, IL-6 and help preferentially drive IgG1, IgA and IgE Ab production by antibody-secreting plasma cells (ASCs) [6-9]. CD8+ precursor T-cells, which maturate into CTLs (cytotoxic T lymphocytes), release antiviral cytokines (IFN-γ) upon recognition of short viral peptides presented by MHC I molecules on virus-infected epithelial cells, and destroy the virus infected cells by exocytosis of cytolytic granules. The granules contain cytolytic protein perforin and granzymes. Perforin is a protein that creates pores in membranes of infected cells. Granzymes are members of serine protease family. In the presence of perforin, granzymes enter into the cytoplasm of infected cells and initiate proteolysis, which triggers destruction of the target cell [10,11]. CTLs could mediate killing of infected cells also by perforin-independent mechanisms of cytotoxicity. This involves binding of Fas receptor in the infected target cell membranes with the Fas ligand (FasL) expressed on activated CTLs. Interaction of FasL with corresponding Fas receptor leads to the activation of caspases, which induce apoptosis in influenza infected cells [12-14].

Unlike T-cells, which recognize linear epitopes presented by MHC molecules, B cells can recognize antigen in its native form. Antibody response against influenza infection is mediated by secretory IgA antibodies and serum IgG antibodies. IgA are transported across the mucosal epithelium of the upper respiratory tract, where they represent the first immunobarrier to influenza viruses. IgG transude from the serum to the mucus by diffusion and are primarily responsible for the protection of the lower respiratory tract [15].

## 3. Protection against influenza infection mediated by antibodies

**Figure 2:**
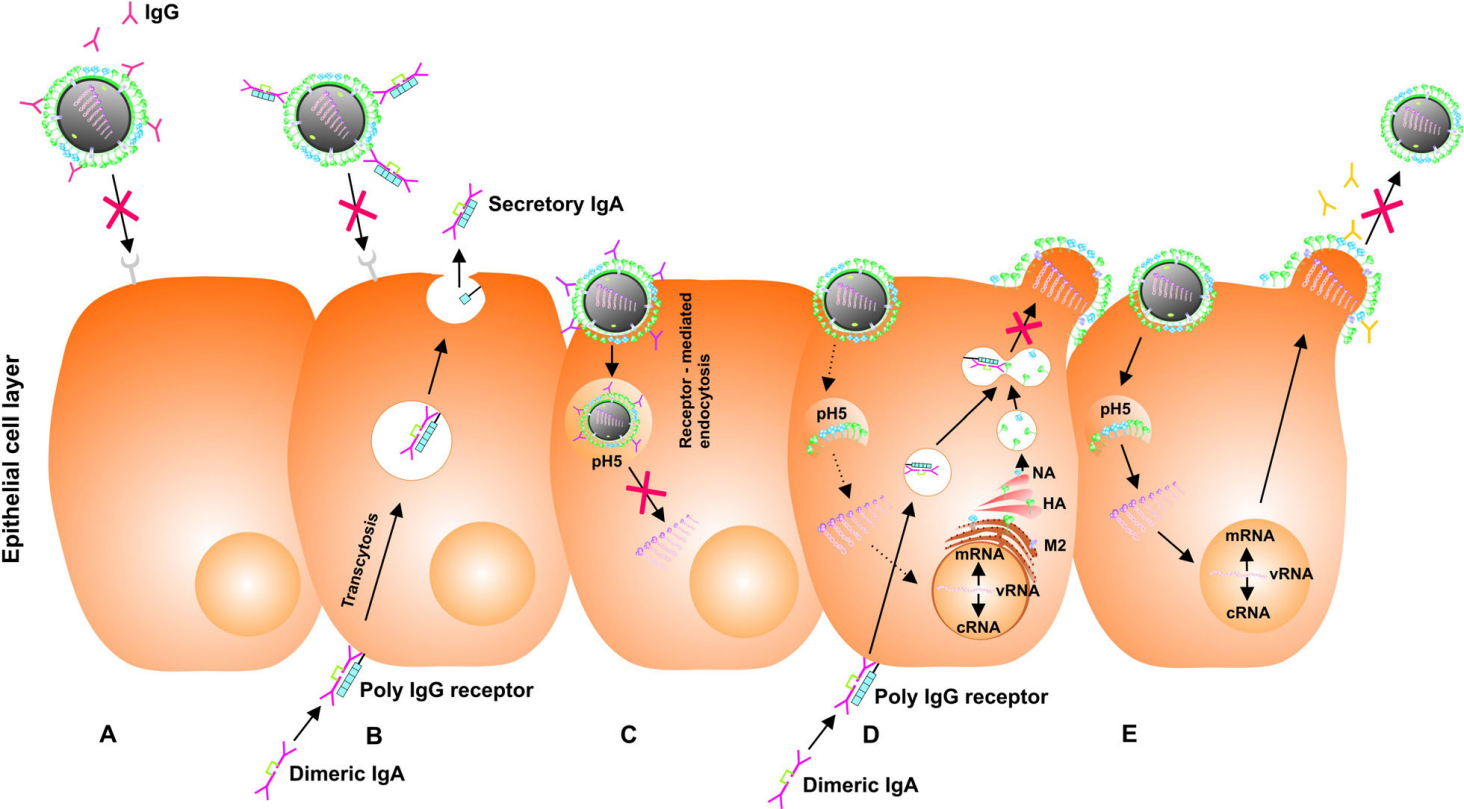
**Mechanisms of antibody-mediated neutralization during influenza infection**. **A. **Serum IgG or **B**. mucosal IgA antibodies specific to hemagglutinin prevent influenza infection by blocking attachment to host cell receptors. **C**. After binding, the virus is internalized by receptor mediated endocytosis. The low pH in the endosome triggers conformational changes in hemagglutinin that expose fusion peptide located in HA2 required for membrane fusion. In this step, antibodies bound to HA2 block the fusion of viral and endosomal membranes and prevent release of ribonucleoprotein complex into the cytoplasm of target cell. **D**. Intracellular neutralization of influenza virus through transcytotic pathway of IgA that complex with viral proteins and inhibit assembly of progeny virions. **E**. Antibodies specific to neuraminidase inhibit release of budding viral particles and further spread of influenza infection by inhibition of neuraminidase activity.

Specific antibodies induced by influenza virus infection can neutralize infection by several different mechanisms (Figure 2). They can directly block virus attachment to the target cells by interfering with virus-receptor interaction and thus prevent influenza infection (Figure 2A). These antibodies are directed to the globular domain of the surface antigen, hemagglutinin [16]. However, because of high variability of influenza A viruses, neutralization activity of these Abs is limited to viral strains, which are antigenically similar to the inducers of Ab production. By contrast, it was shown that mucosal immunity mediated by secreted form of IgA Abs in the upper respiratory tract is more cross-protective against heterologous virus infection than systemic immunity mediated by IgG Abs [17,18]. The strong cross-protective potential of IgA Abs appears to be the consequence of their polymeric nature, resulting in higher avidity of Abs for the influenza virus compared to the monomeric serum IgG Abs [18]. After synthesis by ASC, dimeric IgA (dIgA) Abs bind to the polymeric immunoglobulin receptor expressed on the basolateral surface of the epithelial cells and are transcytosed to the apical surface, where the poly-Ig receptor is cleaved, secretory IgA are released and prevent infection by blocking attachment to the epithelial cells (Figure 2B). Moreover, dIgA Abs are able to bind to the newly synthesized viral proteins within infected cells, thus preventing virion assembly (Figure 2D) [19].

After attachment to the receptor on the target cell, influenza virus is internalized by receptor-mediated endocytosis. Conformational changes of hemagglutinin triggered by the low pH in the endosome activate viral and endosomal membrane fusion. In this step, antibodies, which bind to the non-receptor binding region of HA, could interfere with the low-pH induced conformational change in the HA molecule required for the fusion. Inhibition of the fusion between viral and endosomal membrane proteins mediated by such antibodies prevents release of the ribonucleoprotein complex (RNP complex) into the cytoplasm of the target cell, resulting in the inhibition of viral replication (Figure 2C) [20,21]. In the last step of influenza infection, antibodies specific to the neuraminidase (NA) can bind to budding virus and prevent release of virions from the infected cells. Anti-NA antibodies cause aggregation of virus particles what consequently leads to the reduction of the effective number of infectious units (Figure 2E) [22]. Understanding the processes of antibody-mediated neutralization confers valuable insights into virus-cell interactions and helps to design potent vaccines.

**Figure 3:**
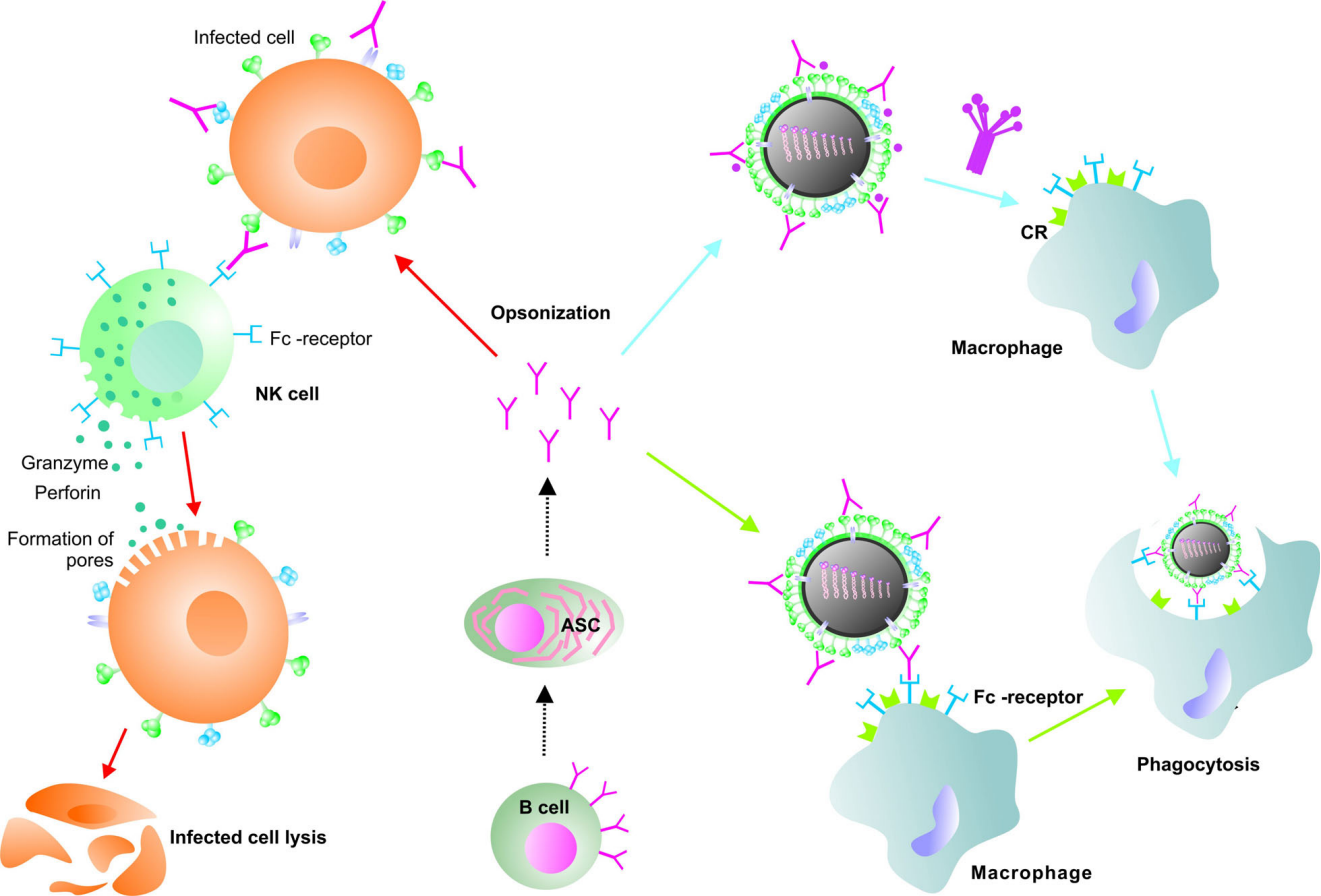
**Indirect anti-influenza mechanisms of protection via Fc region of antibodies**. Infected cells are killed via antibody-dependent cell-mediated cytotoxicity (ADCC) after activation of natural killer cells (NK cell) by Fc region of IgG (red arrow). Phagocytosis of viral particles or infected cells (not shown) is mediated through recognition of Fc region of IgG by macrophages (green arrows) or by interaction of complement with complement receptor on macrophages (CR) (blue arrow).

Recent studies demonstrate that there are also other antibody-mediated mechanisms by which cells infected with influenza virus can be cleared. Antibodies, after coating the infected cells or viral particles, could induce elimination of the virus by FcR-mediated phagocytosis [23] and mediate killing of infected cells via antibody-dependent cell-mediated cytotoxicity (ADCC) or complement-dependent cytotoxicity (CDC) (Figure 3) [24,25]. In the case of ADCC, Fc receptor-bearing natural killer cells (NK cells) recognize Fc region of antibody-coated infected cells and destroy them by releasing cytotoxic granules containing perforins and granzymes, thus limiting the spread of infection [24]. Opsonization of infected cells or free viral particles by specific antibodies could lead to FcR-mediated phagocytosis and subsequent inactivation of the virus in an intracellular compartment of the macrophage [23]. Alternatively, Fc regions of antibodies bound to the surface of infected cells may contribute to the clearance of influenza infection by the activation of classical complement pathway. The interaction of opsonic complement proteins with complement receptor on macrophages (CR) increases the rate of phagocytosis of macrophages, causing direct virolysis or improvement of antibody-mediated inhibition of virus attachment to host cells [26,27]. However, contribution of complement to the protective capacity of antibodies is contradictory, since it was shown that passive transfer of murine polyclonal anti-eM2 serum into C3-negative mice had protective effect [24], while human monoclonal anti-M2 antibodies could not protect complement-depleted mice [25]. It should be noticed that though some antibodies directed to conserved antigens such as M2 do not prevent infection by direct binding to virus, they can contribute to an earlier recovery from the infection by indirect antibody-mediated mechanisms after binding to Fc-receptors on macrophages or NK cells. It is possible that the same mechanism of protection is mediated by antibodies to HA2 glycopolypeptide (HA2 gp), a conserved part of HA. They also do not prevent infection, but their strong protective potential has been proved *in vivo *[28-31]. For this reason understanding the role of the Fc effector function of antibodies in the clearance of influenza infection is required.

## 4. Conserved antigens of influenza virus as inductors of cross-protective humoral immunity

Both, ectodomain of M2 and HA2 gp are conserved antigens inducing antibodies protecting against influenza infection. Therefore, various studies are focused on these two antigens as inductors of heterosubtypic antibody response.

### 4.1 Ectodomain of M2 protein

M2 protein is a single-pass type III membrane protein forming homotetramers representing pH-gated proton channel incorporated into the viral lipid envelope. This proton channel is essential for efficient release of viral genome during viral entry [32]. M2 protein is abundantly expressed at the apical plasma membrane of infected epithelial cells, but only a small number (16-20 molecules/virion) of M2 molecules are incorporated into virions [33,34]. Great attention is paid to the extracellular N-terminal domain of M2 protein (eM2), a 23 amino acid peptide, which is highly conserved in all human influenza A strains. It is therefore an attractive target for preparation of a universal influenza A vaccine. In contrast to hemagglutinin and neuraminidase, eM2 is a weak immunogen [35]. Therefore, various approaches to increase its immunogenicity were used. All of them are based on increasing the immunogenicity of small antigen molecules by insertion of their multiple copies into a suitable immunogen Neirynck et al [36] prepared a fusion protein composed of eM2 and hepatitis B virus core (HBc) protein. This fusion protein has the ability to aggregate into the highly immunogenic virus-like particles inducing a long-lasting protection against lethal influenza A infection. High *in vivo *protective effect of described virus-like particles was proven after intraperitoneal or intranasal immunization of mice and subsequent infection with lethal dose of influenza viruses of various HA subtypes [37]. Efficacy of these particles has been increased by application of new adjuvant CTA1-DD. Combination of the eM2-HBc construct with the new adjuvant led to the protection of mice against lethal infection and a remarkably lower morbidity [38]. Various constructs of eM2 peptide engineered by conjugation to carrier proteins were evaluated as a vaccine, which successfully protected animals against infection with homologous but also heterologous human strains [24,36,37,39-42].

A different approach to increase immunogenicity of eM2 was described by other groups. Constructs composed of four tandem copies of the eM2 peptide fused to flagellin, a ligand of TLR5 (Toll-like receptor 5) [41], or glutathione-S-transferase fusion protein bearing various numbers of eM2 epitope copies [42], were used as immunogens. These studies showed that high eM2 epitope densities in a single recombinant protein molecule resulted in enhanced eM2-specific humoral response and higher survival rates of infected animals.

Another way to stimulate the immune system by small peptide was described by Ernst et al. [39]. They fused the target antigen with hydrophobic protein domain (HD). Such fusion protein can be effectively built into the membrane of small unilamelar liposomes, usually with a diameter of about 100 nm. Ernst et al. [39] prepared liposomes with incorporated recombinant fusion protein eM2-HD comprising three distant aminoacid sequences of eM2 of potentially pandemic strains. Immunization of mice with these eM2-HD liposomes was protective against influenza virus strains of various subtypes and stimulated the production of specific IgG1 antibodies in mouse sera. Moreover, mice passively immunized with these antibodies were protected against lethal infection.

M2 protein in its native state forms a homotetramer, comprising also conformational epitopes, which might play important role in eM2 immunogenicity. It was shown that oligomer-specific antibodies were induced by recombinant eM2 protein mimicking the natural quaternary structure of M2 ectodomain in viral particle [43]. For this purpose, a modified version of leucine zipper from yeast transcription factor GCN4 was bound to eM2. High titers of antibodies recognizing M2 protein in the native conformation were obtained after intraperitoneal or intranasal immunization with this recombinant protein, and immunized mice were fully protected against lethal dose of influenza A virus [43]. Such vaccine could improve quality of humoral immune response with antibodies elicited not only against linear epitopes but also against conformational epitopes.

Above described results indicate that eM2 is a valid and versatile vaccine candidate to induce protective immunity against any strain of human influenza A viruses, and give a promise for finding new "universal" vaccine against flu.

### 4.2 Conserved epitopes of hemagglutinin

Hemagglutinin (HA) is the major influenza virus target antigen recognized by neutralizing antibodies. It is a surface glycoprotein, synthesized as a single polypeptide, which is trimerized. Each monomer of HA is synthesized as a precursor molecule HA0 post-translationally cleaved by host proteases into two subunits, HA1 and HA2 linked by a single disulfide bond [16]. Cleavage into HA1 and HA2 gp is essential for the infectivity of the virus particle and spread of the infection in the host organism [44].

The HA1 of influenza A virus forms a membrane-distal globular domain that contains the receptor-binding site and most antigenic sites recognized by virus-neutralizing antibodies preventing attachment of virus to the host cell. Escape variants with mutation in the antigenic site easily avoid neutralization by existing host antibodies, leading to seasonal influenza outbreaks [45]. In spite of continual antigenic changes of hemagglutinin, common epitopes shared by various strains were identified. Although the degree of sequence diversity between HA subtypes is great, particularly in the HA1 glycopolypeptides, HA2 is its rather conserved part. According to documented results, HA2 has the prerequisite to be one of the potential inductors of protective heterosubtypic immunity [1,28,29,46-48]. HA2 represents the smaller C-terminal portion of hemagglutinin, which forms a stem-like structure that mediates the anchoring of the globular domain to the cellular or viral membrane. N-terminal part of HA2 gp, termed the fusion peptide, plays a substantial role in the fusion activity of influenza virus. It was demonstrated that the rearrangements of HA as well as the fusion process is temperature- and pH-dependent [49,50]. At neutral pH, the N-terminus of the fusion peptide is inserted into the inter-space of HA trimer. At low pH, which triggers the fusion process, N-terminus of the fusion peptide is exposed and inserted into the target membrane, allowing the release of the ribonucleoprotein complex into the cytoplasm [51,16]. Although the epitopes of the HA2 gp are less accessible for interaction with antibodies in native virus than those of HA1 gp, HA2-specific antibodies are induced during natural infection in humans [52] as well as in mice [53]. Significance of HA2-specific antibodies for the heterosubtypic immunity lies in their broad cross-reactivity [1,31,48,54,55]. While HA2-specific antibodies do not act by obstructing the binding of the virus to the host cells [56-58] it should be emphasized that HA2-specific antibodies are able to reduce the replication of influenza viruses of various HA subtypes by several ways: binding of antibody can inhibit the fusion of viral and endosomal membranes [59,60] by preventing the conformation change of HA induced by low pH [20,21,61] or by blocking the insertion of the fusion peptide into the endosomal membrane [62,63]. Moreover, it was shown that passive immunization with monoclonal antibodies against HA2 gp, as well as active immunization with recombinant vaccinia virus expressing chimeric molecules of HA, improve the recovery from influenza infection and contribute to a milder course of infection [28,29]. A recent study showed that increased immunogenicity of HA2 gp could be achieved by unmasking of HA2 gp after removing the highly immunogenic globular head domain of HA1 gp. Headless HA trimers form the conserved HA stalk domain, on which HA2 epitopes are more accessible for B cells than in the native HA. Vaccination of mice with this headless HA immunogen elicited antibodies cross-reactive with multiple subtypes of hemagglutinin and provide protection against lethal influenza virus infection [31].

Hemagglutinin HA1-HA2 connecting region, as well as N-terminal fusion peptide of HA2, are the broadly conserved parts of HA, the latter conserved even among all 16 subtypes of influenza A viruses [1,47,61,64]. Protective potential of the fusion peptide or HA1-HA2 cleavage site of influenza A viruses were investigated by several groups. They found that mice vaccinated with a peptide spanning the HA1-HA2 connecting region exhibited milder illness and fewer deaths upon virus challenge [64,65].

Generation of monoclonal antibodies against universally conserved fusion peptide has attracted interest in the recent past, as such antibodies are known to inhibit the HA fusion activity and to reduce virus replication *in vitro *and also *in vivo *[28,30,54,62,63]. Additionally, passive immunotherapy with Abs reactive with all strains of influenza A could be an alternative for some populations at high risk of infection, like infants, the elderly and the immunocompromised patients, who may not benefit from active vaccination. Several groups described the potential of human monoclonal antibodies against HA2 subunit and its fusion peptide with broad-spectrum protection as a universal passive immunotherapeutic agent against seasonal and pandemic influenza viruses [66-69]. Sui et al. [70] obtained a panel of high-affinity human antibodies that bind to the highly conserved pocket in the stem region of hemagglutinin, comprising part of the fusion peptide and several residues of the HA1 subunit. These antibodies showed a broad degree of cross-reactivity. Moreover, it was suggested that the conformational epitope on HA recognized by one of these neutralizing antibodies (F10) is recalcitrant to the generation of escape mutants [70].

Thus, identification of antibodies against conserved epitopes of hemagglutinin shows the way for their use in passive immunotherapy, designing of antivirals and represents an important step towards development of cross-protective universal vaccine against influenza virus that potentially does not require annual adjustment.

### 4.3 Internal influenza antigens

Nucleoprotein (NP) and matrix protein (M1) of influenza virus are conserved structural influenza antigens, to which antibody response is induced after natural infection. These antibodies, however, do not display a considerable effect on protection against influenza infection [22]. On the other hand, NP, M1 and other inner influenza antigens play important role in the cellular immune response. It was demonstrated that NP- or M1-specific Th cells could augment protective antibody response, aiding the B cells to produce antibodies specific to hemagglutinin [71].

## 5. Conserved antigens of influenza virus as inductors of protective cellular immunity

CTL play an important role in the control of influenza virus infections. They eliminate virus-infected cells, on which surface they recognize foreign antigens derived from endogenously expressed viral antigens presented by MHC class I molecules. Thus, they contribute to the clearance of the virus from the infected tissue and prevent the spread of viral infection. Although CTL do not prevent influenza infection, their beneficial effect on the course of infection was observed after the adoptive transfer of virus-specific CTL clones to mice, resulting in direct lysis of infected cells [72-74]. In addition, depletion of CTL in infected mice led to higher titers of the virus in lungs, increased mortality and more severe disease [75]. Depending on their antigen specificity, CTLs may be subtype-specific or, in case they recognize the internal antigens, they are broadly cross-reactive with various influenza A viral strains. Early studies in mice showed that the majority of influenza-specific CTLs were reactive across subtypes [76,77], what underlines their important role in heterosubtypic immunity. This high crossreactivity is explained by the antigenically conserved targets of CTL represented mostly by inner influenza antigens (e.g. NP, M1 and PB1, PB2) [78-81]. However, some conserved T-cell epitopes were identified also on variable surface influenza antigens [82-84].

Recent data support the beneficial role of T-cell response in reducing the severity of infection also in humans [85-88]. Additionally, cross-reactive CTLs recognized different subtypes of influenza A virus and their protective effect was shown also in individuals, who did not have specific antibodies against a given influenza virus they were exposed to [89]. Therefore, vaccination strategies focused on generating T-cell-mediated immune responses directed towards conserved epitopes of influenza virus are also considered.

### 5.1 Conserved influenza virus T-cell epitopes identification and their vaccine application

T-cell epitopes are intensively studied as an alternative to the current vaccine strategy based mainly on the induction of the strain specific virus-neutralizing antibodies. Identification of conserved CTL epitopes shared by many influenza strains could represent the basis of vaccination strategies. This approach would be beneficial in the case of annual influenza epidemic and a potential pandemic, when humoral immunity is poorly or not protective due to the absence of pre-existing antibodies against emerging strains in the population [90,91].

While CTL mediated immunity is considered to be weak, epidemiological data indicate induction of cross-protective immunity in humans, who overcame influenza infection in the past [85]. It was shown that memory T-cells against the conserved epitopes confer protection from the infection with the virus strains of different subtypes in humans [82,85,86,88,89,92,93].

Studies in mice demonstrated that, similar to the live influenza vaccine, adenovirus-based vaccine and DNA immunization induced CTL cross-protective immune response against infection with multiple influenza A subtypes [94-98]. The variable rate of cross-reactive CTL response was achieved also by using adjuvants, or various formulations and delivery systems with experimental influenza vaccines in preclinical animal studies [reviewed in [99]]. It was shown that application of virus-like particles or virosomal vaccines could be successfully used for efficient delivery of multiple CTL epitopes to the target cells resulting in induction of CTL response [100,101].

Heterosubtypic immunity mediated by CTLs was described in naturally infected humans [88,89,102]. It is developed mainly against conserved epitopes of NP, M1 and NS1 [82,103-106]. Kreijtz et al. showed that virus-specific CTL developed in humans as a response to previous exposition to seasonal influenza A viruses of the H3N2 and H1N1 subtypes displayed considerable cross-reactivity also with avian influenza viruses (e.g. A/H5N1) [86]. Thus, it could be supposed that obtained pre-existing T-cell immunity in humans may help to decrease the severity of infection during a pandemic outbreak in comparison to those individuals, who lack cross-reactive influenza specific CTL populations [86,88,107]. Therefore, vaccines based on conserved CTL epitopes represent a reasonable approach to generate effective broadly protective cellular immunity against influenza viruses of various subtypes.

## 6. Immunodominance of influenza T-cell epitopes

To develop vaccines capable of stimulating effective T-cell response, it is necessary to understand the factors contributing to the immunodominance of CTL epitopes. During viral infection, a large number of peptides are generated by processing of viral proteins in the proteasomes of infected cells. Only a small fraction of these peptides are presented by MHC class I molecules and subsequently recognized by specific CTL. This hierarchy of CTL response proved in animals [108] and in humans [104] is called immunodominance. There are several factors, which contribute to this phenomenon: HLA haplotype and its binding affinity to individual epitopes, repertoire of T-cell receptors, processing and presentation of viral peptides and interaction of CTL with antigen-presenting cells [109,110]. It was shown that efficiency of epitope processing is one of the dominant factors affecting immunogenicity of multi-epitope vaccine [111,112].

The most frequently used models for such immunological studies are inbred mice, like B57BL6 (H-2^b^) or BALB/c (H-2^d^) mice. Therefore, T-cell influenza specific epitopes in inbred mice were studied by many authors. Comprehensive analysis regarding existing influenza A epitopes in mice among avian and human influenza strains was done by Bui et al. [113]. However, not all T-cell epitopes are equally immunogenic. In inbred mice B57BL6 (H-2^b^), peptides from nucleoprotein D^b^NP_366-374 _and from a subunit of viral RNA polymerase D^b^PA_224-233 _are immunodominant, while nucleoprotein epitope K^d^NP_147-155 _is immunodominant in BALB/c (H-2^d^) mice [84,114-116].

In contrast to inbred mice, the search for CTL epitopes suitable for development of CTL epitope-based vaccine in humans is more complicated [113]. The main reason is that HLA genes in humans are extremely polymorphic. Therefore, the knowledge of HLA restriction in population, which will be vaccinated, is necessary. The complexity of HLA molecules could be reduced by clustering them into sets of molecules that bind largely overlapping peptides. Such clustering was introduced by Sette and Sidney in 1999. They defined HLA supertypes as a set of HLA molecules that have similar peptide binding motifs and overlapping peptide binding repertoires [117]. Nine different supertypes (A1, A2, A3, A24, B7, B27, B44, B58, B62) were defined on the basis of their specifity for the main anchor positions of presented peptides. Later, other three HLA I supertypes (A26, B8 and B39) were described by Lund et al. [118]. Recent analysis provided an update of HLA I alleles classification into supertypes and is expected to facilitate epitope identification and vaccine design studies [119].

An example of most frequently recognized conserved epitopes of influenza antigens in humans represents M1_58-66 _CTL epitop, which is restricted by the high prevalence allele HLA-A*0201 and could be a promising vaccine candidate [120]. Computer programs available today can predict binding epitopes of a given protein for the most common HLA allele [121,122]. *In silico *analysis supports the proposition that the T-cell response to cross-reactive T-cell epitopes induced by vaccination or seasonal viral exposition may have the capacity to attenuate the course of influenza infection in the absence of cross-reactive antibody response [123,124]. The ability to predict the CTL epitope immunogenicity and recognition patterns of variant epitopes enhances the probability of the optimal selection of potential targets of immune response and can be utilized for vaccine design [93,113,125]. In spite of the differences in various classification schemes, the concept of HLA supertypes has been effectively used to characterize and identify promiscuously recognized T-cell epitopes from a variety of different disease targets, as are those of hepatitis C virus [126,127], SARS [128] or HIV [129,130] but also influenza virus [131].

A critical requirement for CTL epitope-based strategy is to identify and select promiscuous CTL epitopes that bind to several alleles of HLA supertypes to reach maximal population coverage. The utilization of supertype-restricted epitopes, which bind with significant affinity to multiple related HLA alleles, provides solution to this problem [117]. As described before, 80-90% population coverage can be achieved in most prominent ethnicities by focusing on only three major HLA class I supertypes -A1, -A3 and -B7 [132,133]. By including two additional supertypes (A1, A24), 100% population coverage in all major ethnicities could be reached [117,132]. Recently, HLA class I -A2, -A3 or -B7 supertype-restricted epitopes conserved among different viral subtypes of influenza virus were identified, what could be of relevance for the development of a potential supertype-restricted, multiepitope CTL-based vaccine protective against any subtype of influenza virus [82,103,113,134].

## 7. Conclusion

One of the drawbacks of currently available inactivated vaccines is the lack of broad cross-protective humoral and cell-mediated immune response against any influenza virus. Their efficacy is limited due to the genetic variation of influenza viruses. Therefore, their annual reformulation is necessary in an attempt to antigenically match the currently circulating strain for each of the three vaccine strains or their subunits (HA and NA of H1N1 and H3N2 of influenza A virus as well as of influenza B virus) from which they are composed. Increasing amount of information about conserved epitopes of influenza viruses brings us closer to the development of the universal vaccine. Such vaccine should contain both, conserved B-cell epitopes that are important for induction of cross-protective antibodies and CTL epitopes for the involvement of the cellular arm of the immune response to the overall protective effect [90]. It was shown that the pre-existing memory T-cell immunity as defense against seasonal influenza strains may have the capacity to moderate the course of disease in the case of newly emerging flu viruses in the absence of cross-reactive antibody response [86,93,123,124]. It was also shown that it would be possible to elicit the CTL response simultaneously directed against multiple supertype-restricted conserved CTL epitopes [135-139]. This could be relevant for the development of a potential supertype-restricted multiepitope CTL based vaccine, with the effort to reach wide population coverage. Even though recent reports support a beneficial role of T-cell response in reducing human infections [86-88,124], there are still many questions regarding the feasibility of designing an effective supertype-restricted CTL epitope based vaccine in humans. In addition to CTL epitopes, B-cell epitopes from conserved influenza antigens that can elicit cross-protective humoral response should also be considered as a component of novel vaccines. Recently, highly cross-reactive monoclonal antibodies directed against conserved epitopes of HA2 subunit, including fusion peptide, were identified [28,30,66,68-70]. HA2 subunit region as well as M2 protein are promising candidates for design of vaccine constructs aimed at providing broad-spectrum immunity to influenza viruses [1,28,31,37,45]. Cross-protective potential of HA2 and eM2 could be increased by optimization of their delivery and immunogenicity using vaccine vectors that target multiple Toll-like receptors for efficient stimulation of innate immunity and subsequent enhancement of the adaptive immune response [41,140]. Conserved B- and T-cell epitopes, thus, could represent the basis for preparation of universal vaccine and bring new hope for development of pandemic or universal influenza vaccine.

## Competing interests

The authors declare that they have no competing interests.

## Authors' contributions

Both authors contributed to the original draft manuscript and approved the final version. The fine art of all figures was designed by ZS.
